# Preventing stillbirth from obstructed labor: A sensorized, low-cost device to train in safer operative birth

**DOI:** 10.3389/fgwh.2022.1039477

**Published:** 2023-01-30

**Authors:** Shireen Jaufuraully, Carmen Salvadores Fernandez, Biswajoy Bagchi, Priya Gupta, Adrien Desjardins, Dimitrios Siassakos, Anna L. David, Manish K. Tiwari

**Affiliations:** ^1^ Wellcome/EPSRC Centre for Interventional and Surgical Sciences, University College London, London, United Kingdom; ^2^ Elizabeth Garrett Anderson Institute for Women’s Health, University College London, London, United Kingdom; ^3^ Nanoengineered Systems Laboratory, Mechanical Engineering, University College London, London, United Kingdom; ^4^ Department of Medical Physics and Biomedical Engineering, University College London, London, United Kingdom; ^5^ National Institute for Health Research (NIHR) University College London Hospitals Biomedical Research Centre (BRC), London, United Kingdom

**Keywords:** stillbirth, obstructed labor, cesarean section, sensorized glove, operative vaginal birth

## Abstract

**Background:**

98% of stillbirths occur in low- and middle- income countries. Obstructed labor is a common cause for both neonatal and maternal mortality, with a lack of skilled birth attendants one of the main reasons for the reduction in operative vaginal birth, especially in low- and middle- income countries. We introduce a low cost, sensorized, wearable device for digital vaginal examination to facilitate accurate assessment of fetal position and force applied to the fetal head, to aid training in safe operative vaginal birth.

**Methods:**

The device consists of flexible pressure/force sensors mounted onto the fingertips of a surgical glove. Phantoms of the neonatal head were developed to replicate sutures. An Obstetrician tested the device on the phantoms by performing a mock vaginal examination at full dilatation. Data was recorded and signals interpreted. Software was developed so that the glove can be used with a simple smartphone app. A patient and public involvement panel was consulted on the glove design and functionality.

**Results:**

The sensors achieved a 20 Newton force range and a 0.1 Newton sensitivity, leading to 100% accuracy in detecting fetal sutures, including when different degrees of molding or caput were present. They also detected sutures and force applied with a second sterile surgical glove on top. The software developed allowed a force threshold to be set, alerting the clinician when excessive force is applied. Patient and public involvement panels welcomed the device with great enthusiasm. Feedback indicated that women would accept, and prefer, clinicians to use the device if it could improve safety and reduce the number of vaginal examinations required.

**Conclusion:**

Under phantom conditions to simulate the fetal head in labor, the novel sensorized glove can accurately determine fetal sutures and provide real-time force readings, to support safer clinical training and practice in operative birth. The glove is low cost (approximately 1 USD). Software is being developed so fetal position and force readings can be displayed on a mobile phone. Although substantial steps in clinical translation are required, the glove has the potential to support efforts to reduce the number of stillbirths and maternal deaths secondary to obstructed labor in low- and -middle income countries.

## Introduction

Worldwide, there are almost 2.6 million stillbirths per year, and low-and-middle-income countries (LMICs) make up a staggering 98% of these (1). 50% of stillbirths occur in labor (2). Obstructed labor is responsible for up to 70% of perinatal deaths (3), with malpresentation and malposition accounting for nearly a third of all cases. In competent hands, operative vaginal birth (OVB), such as Ventouse, forceps, or Kiellands forceps delivery, is a safe and quick procedure that can overcome obstructed labor due to malposition. It also reduces the chance of second stage cesarean section (CS). However, OVB is globally in decline, with a lack of skilled birth attendants and staff training cited as one of the main reasons for the reduction in OVB in LMICs (4). In untrained hands, OVB can be dangerous for both the mother and fetus. Instrument placement is poor in nearly 30% of cases, leading to trauma and extended hospital stays (5). Ultrasound scan can be used to ascertain fetal position in the second stage of labor (6), but is expensive and requires further staff training. There is an unmet need for a low-cost device that could help birth attendants identify malposition and obstructed labor earlier, and thus seek medical care earlier. There is also a need to facilitate training in, and safe OVB, and thus reduce the burden of stillbirths secondary to obstructed labor.

Recent advances in the work mimicking the human haptic system have gained tremendous appeal (7, 8). The way in which human beings respond to external stimuli—which is processed through the haptic sensory system—is based on personal perception. This can lead to human error and difficulties when teaching or training to carry out certain tasks, such as surgical interventions and other medical procedures in which small deviations can lead to life-threatening issues. Being able to mimic said receptor network and sensory systems with the use of sensors can lead to obtaining quantifiable data which may lead to improved outcomes in medical procedures, serving as aiding tools for clinicians. One of the strategies followed to mimic the haptic system has revolved around the design and creation of sensorised gloves which are able to monitor and track physical activity for rehabilitation purposes (9). The sensors chosen are inspired by the haptic system, and aim to incorporate both cutaneous and kinaesthetic components. The combination of both position and force sensors using the technology described leads to further information obtained by the smart gloves (10), and this, together with strain and flexural sensors (11–14), further builds towards the idea of slowly mimicking the functions and sensing abilities of the skin, moving a step at a time closer to its vast complexity (15). It is becoming increasingly recognized that another essential requirement in this field surrounds the use of thin sensors: the haptic perception of the clinician must not be altered by their inclusion in the device (16). This has led to growing interest in virtual reality gloves for the same rehabilitation purposes previously mentioned (17) using image recognition strategies, slowly advancing towards human-machine interface related work (18). This all has inevitably raised awareness regarding the importance of acquired data, its analysis, storage and display (19). Work has also been targeted towards the development of algorithms to extract relevant information from this data in a surgical environment (20).

With the above in mind, the aims of the study were to develop a low-cost, sensorized surgical glove to aid in ascertaining fetal position and force applied during digital vaginal examination, with particular focus on ensuring that the sensors were thin enough to avoid disruption to the clinician's own haptic feedback. The glove was designed so that it could detect fetal sutures and force applied while also maintaining sterility by having a second sterile surgical glove covering it. Further aims were to design a user-friendly interface that could display data and alert clinicians effectively, as well as developing a wireless node and smartphone app for ease of use and universalization. The above features lend to a device that can be used in low resource settings to aid training and practice in safe OVB.

## Materials and methods

### Glove fabrication, calibration, and app setup

**Figure 1:**
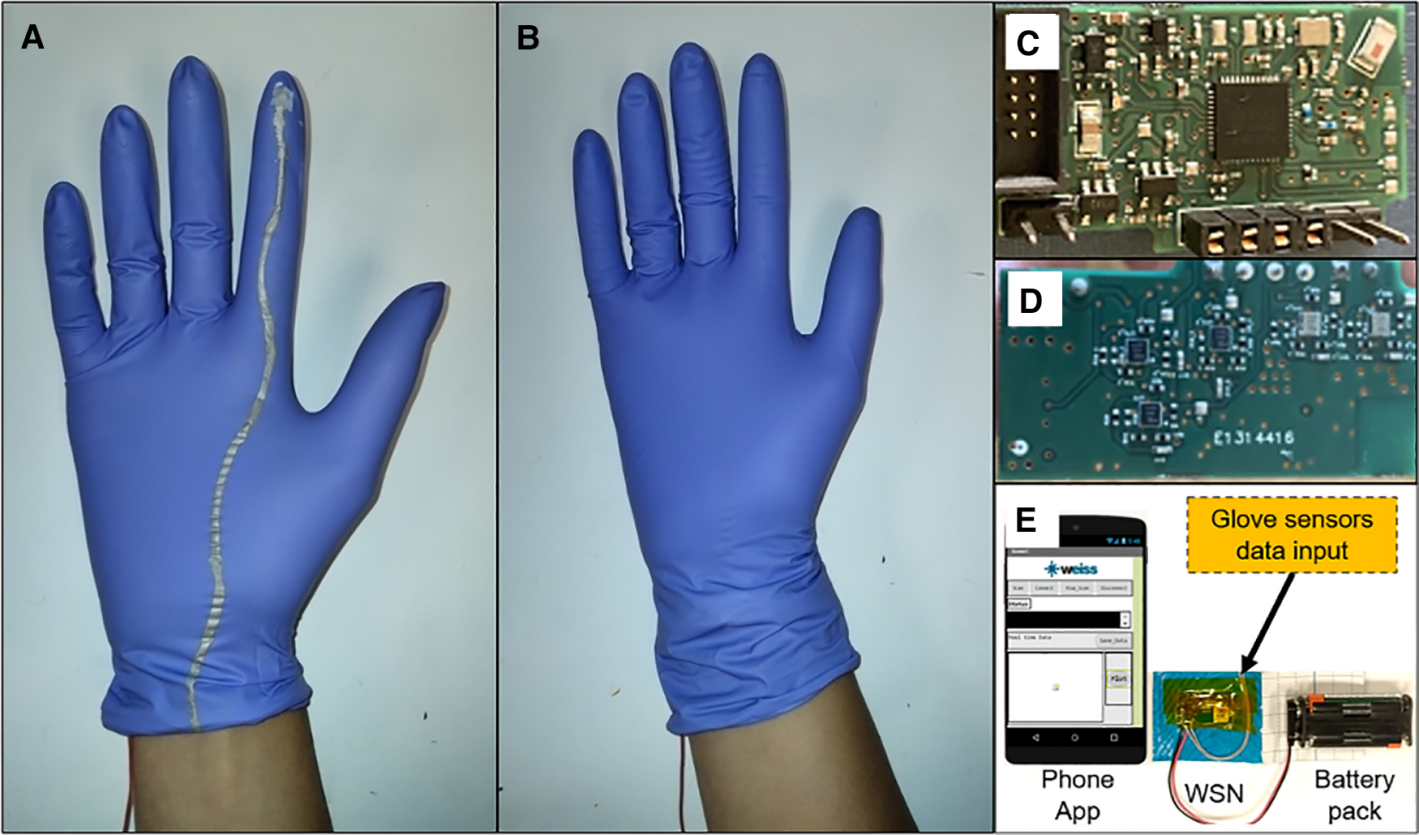
Sensorised device for digital vaginal examination. (**A**) Photograph of sensorised glove with sensor on the fingertip and flexible interconnect printed on the glove. (**B**) Photograph of sensorised glove covered by a sterile surgical glove for sterility purposes. (**C**) Photograph of customized wireless sensor node (front). (**D**) Photograph of customized wireless sensor node (back). (**E**) Designed wireless system for real-time data display.

The device consists of flexible pressure and force sensors mounted onto the fingertips of a surgical glove, and flexible interconnects which were directly printed on the glove (Figure 1A). The sensors were integrated by simply spray coating a metal-oxide nanocomposite onto the fingertip of the glove. The sensors work on the principle of triboelectricity (21), which is a type of contact electrification which occurs when two materials come into contact or are rubbed against each other. This contact electrification coupled with electrostatic induction is what enables the sensors to produce a current when making contact/rubbing against a material. The sensors and interconnects were tested on surgical gloves of the following typical materials: latex, nitrile, polyisoprene and vinyl. The total cost of materials used to fabricate the sensors and interconnects is less than US$1 per glove. For sterility purposes, the glove was covered by a standard sterile surgical glove routinely used in practice (Figure 1B).

A customized wireless sensor node (WSN) was also designed to extract real-time data from the sensorized glove (Figures 1C, D). This WSN is based on a 4-layer printed circuit board (PCB) that includes an nRF51 Bluetooth module (BLE4.0), with an analog signal-conditioning front-end circuitry (for triboelectric sensors) to deal with the signal dynamic range during wireless data transmission. The WSN connects to a phone app developed for real-time data display on the mobile phone screen. The app was developed using the MIT App Inventor platform. This platform has the functionality to create a gateway between the two hardware devices (mobile phone and WSN) and to create a mobile app. This mobile app shows the data converted and transmitted by the customized WSN. The real-time data is displayed on a mobile phone screen through the app. The app also saves this real-time data for further analysis (Figure 1E).

**Figure 2:**
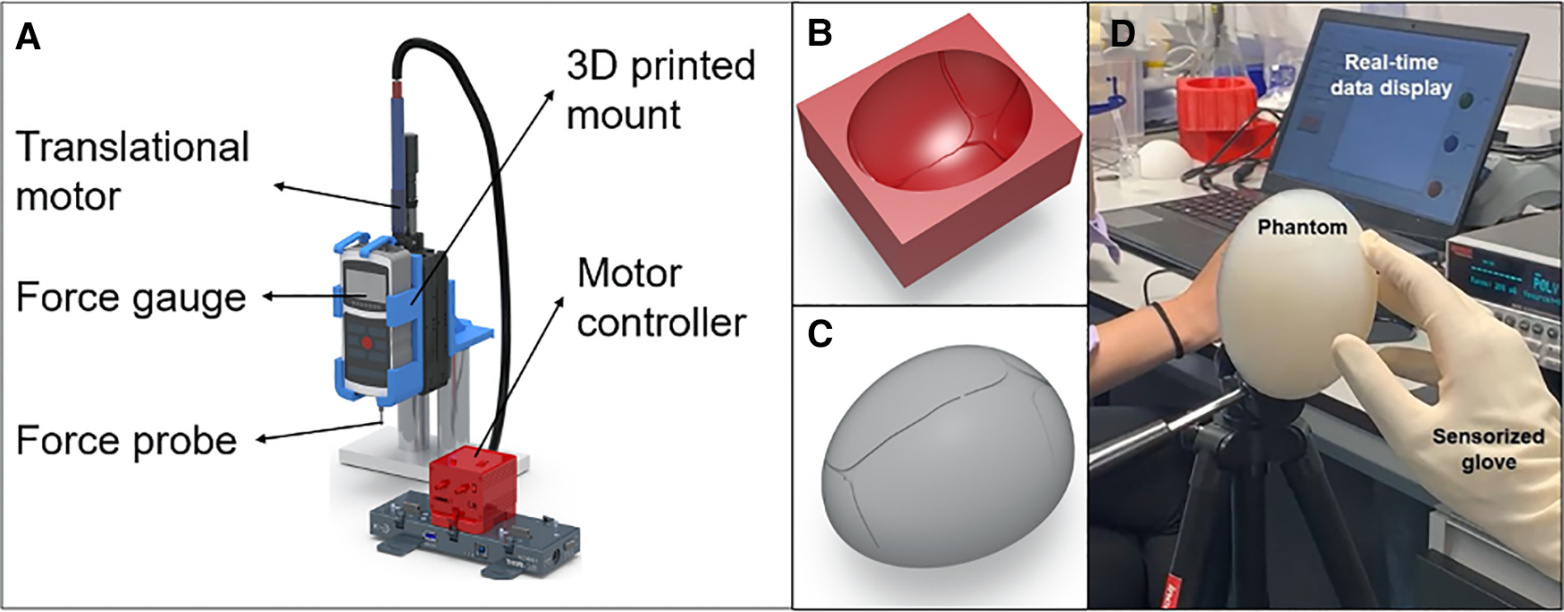
Test set up in the lab. (**A**) Schematic representation of the force calibration setup. (**B**) Mold created to fabricate phantom models of the fetal head (**C**) Schematic representation of the fetal head phantom model created. (**D**) Test set up used to validate the sensors.

The sensors were calibrated using a setup (Figure 2A) that combines a motorized translation stage (PT1-Z8, Thorlabs), a force gauge (M5-5 Mark-10) and an electrometer (Keithley 6517B) connected to a laptop and working simultaneously by means of a virtual interface (VI) developed with custom software (LabView). The setup achieves a 10 micrometer step size (the minimum distance between two points that can be recorded) and a 0.0088 Newton (N) sensitivity, which enables detection of different stiffnesses (resistance to elastic deformation) through concomitant force and displacement readings on materials of known elastic moduli. Through this setup, calibration curves for each sensor can also be obtained in order to relate current measurements with force values (Supplementary Information S1). The sensors were mounted underneath the force probe and connected to the electrometer to obtain the current values and (Figure 2A).

### Development of fetal head phantoms

In order to test and validate the sensors, a test setup was created in-lab. A model of the fetal head was developed to replicate skull sutures in term pregnancy (Figures 2B, C). Molds were developed using Altair Inspire software and PLA 3D printed using the Ultimaker 3D printer. Ecoflex, a type of silicone elastomer, was poured inside the molds and cured for 2 h at 80 °C.

### Testing of the sensor glove

An obstetrician with 6 years' experience tested the glove on the fetal models by performing a mock vaginal examination at full dilatation, to demonstrate repeatability and accurate detection of fetal sutures. The obstetrician wore the sensor glove and covered it with a sterile surgical glove and simply rubbed through a given suture on the fetal head phantom mounted onto a tripod (to mimic the angle of a fetal head during vaginal examination) 100 times. The sensors were connected to an electrometer (Keithley 6517B) which was connected in turn to the laptop and data was displayed in real-time and recorded by means of a virtual interface created with custom software (LabView) (Figure 2D). Signals from the sensorized glove were recorded and analyzed.

### Patient and public involvement

Throughout the design of the device, the team ran five patient and public involvement (PPI) groups over the course of 18 months, where women and birthing people from minority backgrounds, and those with previous experience of operative birth, were invited to hear about, and give feedback regarding, the sensorized glove.

## Results

### Detection of sutures

**Figure 3:**
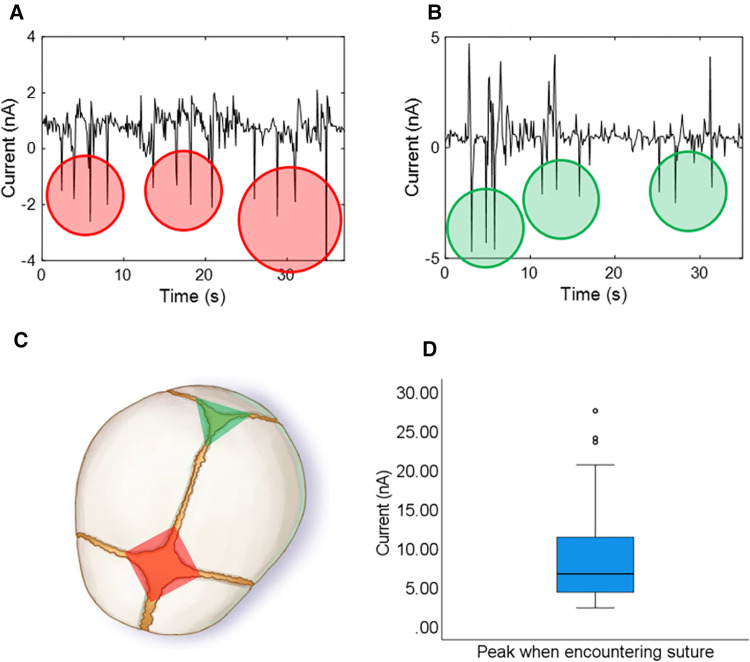
Current signal results using the glove to examine skull sutures, and the anterior and posterior fontanelle on the phantom (each peak corresponds to a crossed suture and peaks are counted to distinguish fontanelles – 4 peaks/sutures for the anterior fontanelle and 3 peaks/sutures for the posterior fontanelle). (**A**) Anterior fontanelle results. (**B**) Posterior fontanelle results. (**C**) Schematic representation of the fontanelles and sutures of a fetal head. (**D**) Boxplot showing the amplitude of the current peaks produced when crossing a suture (100 repetitions).

Due to the 0.1 N sensitivity achieved, the sensors produce distinctive current peaks when crossing a suture, as shown in Figures 3A, B. The anterior fontanelle (highlighted in red in Figure 3C) is diamond shaped and associated with four sutures (frontal, coronal on either side, and sagittal), hence why four current peaks are created as the sensor passes over the four sutures (Figure 3A). The posterior fontanelle (highlighted in green in Figure 3C) is triangle shaped and associated with three sutures (lambdoid on either side and sagittal), hence why three current peaks are created when passing over the three skull sutures (Figure 3B).

The boxplot in Figure 3D shows the amplitude of the current peaks produced when crossing a skull suture throughout the 100 repetitions as part of our test protocol to ensure accuracy and repeatability. The magnitude of the peaks only surpassed a threshold of 1.7 nA when crossing a suture and at no other time, thus showing 100% sensitivity and 100% specificity in detecting sutures. By counting the skull sutures (number of peaks above the threshold value), a user-friendly interface was developed using custom software (LabView) to display either a green triangle (when encountering the posterior fontanelle) or a red diamond (when encountering the anterior fontanelle) to visually represent fetal position (Figure 3C and Supplementary Video S1).

Furthermore, the sensors worked with multiple glove materials, namely: latex, nitrile, polyisoprene and vinyl surgical gloves, and therefore are safe to use in those with particular allergies. They can accurately detect fetal skull sutures and objectively detect fetal position, even when a sterile surgical glove is placed on top of the device.

### Quantification of contact force

The calibrated sensors achieved a 20 N force range (Supplementary Figure S1). The user-friendly interface described above allowed a force threshold to be set manually, alerting the clinician when excessive force has been applied to the fetal head by means of a traffic-light colour system which changes colour when the manually set thresholds are surpassed (Supplementary Video S2).

### Smartphone app

The sensors have successfully interfaced with a WSN and data has been effectively displayed in real-time on the developed mobile phone app. The wireless node allows ease of manipulation, robustness and continued use to test the different set of data signals obtained from the sensorized glove (SupplementaryVideo S3).

### Patient and public involvement

Patient and public involvement panels have welcomed the device with great enthusiasm. Group numbers ranged from six to 30, and feedback was gained on the design and functionality of the glove. Over 90% of women indicated they would want to be examined with the glove in labor if it could improve safety outcomes for their babies, and aid in decision making regarding mode of birth (Video links in Supporting Information 3).

## Discussion

Under phantom conditions to simulate the fetal head in labor, the novel sensorized glove can accurately determine fetal sutures and provide real-time force readings, even with a second layer of sterile surgical glove on top, ensuring sterility and safety. It is low cost—it is estimated that the materials to create the sensorized glove costs less than $1 and it may be lower when scaled to larger (production) volumes. Building on the phone app developed can lead to the creation of an app-based user-friendly interface such as the one developed for the laptop, which may lead to universalization. It would thus lead to the replacement of expensive equipment, and it would allow anyone to simply view the displayed data (in the form of visual indications such as the traffic-light system mentioned above) on a mobile phone. The measured results show that the operating voltage of this designed WSN is 3 V, the total power consumption is approximately ∼24 mW, and the Bluetooth data transmission distance is 10 meters.

To our knowledge, this is the first study involving the development and use of a sensorized glove to detect fetal head position and force exerted on the fetal head during vaginal examination. The strengths in this study lie in the calibration of the sensors and multiple examinations to demonstrate 100% accuracy. The main limitation is that phantoms of the fetal head were used rather than testing the glove *in vivo*, but these were the first steps in clinical translation. Other studies in obstetrics have focused solely on glove-mounted pressure sensors. A few studies with sensorised gloves have also followed the patterns and directions previously described, although remain widely unexplored. Sensors have been incorporated into gloves to measure forces applied to turn a fetus during external cephalic version (22, 23), to compare forces imposed on the fetus in vaginal vs. caesarean delivery (24), and forces exerted in the context of shoulder dystocia (25). These studies demonstrated clinical utility and an insight into the forces applied in the obstetrics context. Nevertheless, the approach we have chosen, and which appears to be a gap in the relevant surgical literature, is to build on the work done on surgical smart gloves but purposely targeting its use as assistive technology for clinicians in addition to monitoring or assessment of forces applied. There is currently no data on what constitutes a “safe” force to apply during vaginal examination, as no such device to measure force applied has existed up to now. However, study data from fetal trauma during impacted cesarean section suggests that undue force can result in skull fractures in 1% of deliveries (26), highlighting that such a device could be used to collect such force data and aid in training to reduce fetal trauma.

Our study is unique as we have developed a smartphone app which provides information that the birth attendant can act upon, and therefore has the potential to change clinical outcomes as a direct result of the data displayed. Furthermore, we also aim to expand on the “cutaneous” capabilities of these previous studies not only to provide force measurements, but to also give further information such as changes in stiffness. This way we aim to progress and contribute towards more comprehensive mimicking of the human tactile system by progressively adding capabilities (not just force feedback).

It is hoped that the glove will have a similar potential and impact to the CRADLE (Community blood pressure monitoring in Rural Africa & Asia: Detection of underLying pre-Eclampsia and shock) Vital Signs Alert device, which is a low cost, accurate device to measure vital signs in low-resource settings. It has been developed for use in pregnancy and instances of pre-eclampsia and shock, and measures blood pressure, heart rate, and calculates the shock index in women with postpartum haemorrhage, The device allows earlier recognition of shock and identifies those that require transfer to a tertiary setting (27). Similarly, we envisage that the glove will help the wearer identify obstructed labor and malposition earlier, and thus allow earlier transfer for medical care. This is particularly pertinent when a woman can be in obstructed labour for days, and also be left with the devastating consequences of obstetric fistula formation; in a study of over 4,000 women presenting with obstetric fistula in East and Central Africa, 84% had suffered from a stillbirth (28). The glove would be a welcome intervention in potentially recognising obstructed labor earlier to reduce both neonatal and maternal morbidity and mortality.

The next steps include clinical translation of the glove *via* a study on laboring women and neonates to ensure it can detect fetal head position *in vivo*. It is aimed that the glove will tackle several challenges faced by LMICs with regards to stillbirth secondary to obstructed labor—by supporting safer clinical training, practice in OVB, and decision-making regarding mode of birth. With the advent of telemedicine and development of the smartphone app, in time, it is anticipated that clinicians from around the world will be able to access real-time data regarding fetal position and other examination findings remotely, and be able to provide senior clinical input and expertise to those in need and in remote areas.

## Data Availability

The raw data supporting the conclusions of this article will be made available by the authors, without undue reservation.

## References

[1] Frøen JF Friberg IK Lawn JE Bhutta ZA Pattinson RC Allanson ER Lancet Ending Preventable Stillbirths Series study group. Stillbirths: progress and unfinished business. Lancet. (2016) 387(10018):574–86. 10.1016/S0140-6736(15)00818-1 26794077

[2] Lawn JE Blencowe H Waiswa P Amouzou A Mathers C Hogan D Lancet Ending Preventable Stillbirths Series study group; Lancet Stillbirth Epidemiology investigator group. Stillbirths: rates, risk factors, and acceleration towards 2030. Lancet. (2016) 387(10018):587–603. 10.1016/S0140-6736(15)00837-5 26794078

[3] Ayenew AA. Incidence, causes, and maternofetal outcomes of obstructed labor in Ethiopia: systematic review and meta-analysis. Reprod Health. (2021) 18:61. 10.1186/s12978-021-01103-0 33691736 PMC7944638

[4] Bailey PE van Roosmalen J Mola G Evans C de Bernis L Dao B. Assisted vaginal delivery in low and middle income countries: an overview. BJOG. (2017) 124(9):1335–44. 10.1111/1471-0528.14477 28139878

[5] Ramphul M Kennelly MM Burke G Murphy DJ. Risk factors and morbidity associated with suboptimal instrument placement at instrumental delivery: observational study nested within the instrumental delivery & ultrasound randomised controlled trial ISRCTN 72230496. BJOG. (2015) 122(4):558–63. 10.1111/1471-0528.13186 25414081

[6] Ghi T Eggebø T Lees C Kalache K Rozenberg P Youssef A ISUOG Practice Guidelines: intrapartum ultrasound. Ultrasound Obstet Gynecol. (2018) 52:128–39. 10.1002/uog.19072 29974596

[7] Al-Handarish Y Omisore OM Igbe T Han S Li H Du W A survey of tactile-sensing systems and their applications in biomedical engineering. Adv Mater Sci Eng. (2020) 2020:4047937. 10.1155/2020/4047937. [Epub ahead of print]

[8] Li F Wang R Song C Zhao M Ren H Wang S A skin-inspired artificial mechanoreceptor for tactile enhancement and integration. ACS Nano. (2021) 15(10):16422–31. 10.1021/acsnano.1c05836 34597014

[9] Pompili G Baldi TL Barcelli D Prattichizzo D. Development of a low-cost glove for thumb rehabilitation: design and evaluation. Proc 2020 IEEE int conf human-machine syst ICHMS 2020. (2020:0-6).

[10] Henderson J Condell J Connolly J Kelly D Curran K. Review of wearable sensor-based health monitoring glove devices for rheumatoid arthritis. Sensors. (2021) 21(5):1–32. 10.3390/s21051576 PMC7956752 33668234

[11] Oess NP Wanek J Curt A. Design and evaluation of a low-cost instrumented glove for hand function assessment. J Neuroeng Rehabil. (2012) 9(1):2. 10.1186/1743-0003-9-2 22248160 PMC3305482

[12] Chen X Gong L Wei L Yeh S Zheng L Zou Z. System with soft gloves. IEEE Trans Ind Informatics. (2021) 17(2):943–52. 10.1109/TII.2020.3010369

[13] Chen X Gong L Zheng L Zou Z. Soft exoskeleton glove for hand assistance based on human-machine interaction and machine learning. Proc 2020 IEEE int conf human-machine syst ICHMS 2020. (2020). 10.1109/ICHMS49158.2020.920938

[14] Ben-tzvi P Ma Z. Sensing and force-feedback exoskeleton (SAFE). IEEE Trans Neural Syst Rehabil Eng. (2015) 23(6):992–1002. 10.1109/TNSRE.2014.2378171 25494512

[15] Mohan A Devasahayam SR Tharion G George J. A sensorized glove and ball for monitoring hand rehabilitation therapy in stroke patients. Proc - 2013 Texas instruments India educ conf TIIEC 2013. (2013). p. 321–327.

[16] Lee S Franklin S Hassani FA Yokota T Nayeem MOG Wang Y Nanomesh pressure sensor for monitoring finger manipulation without sensory interference. Science. (2020) 370(6519):966–70. 10.1126/science.abc9735 33214278

[17] Placidi G. A smart virtual glove for the hand telerehabilitation. Comput Biol Med. (2007) 37(8):1100–7. 10.1016/j.compbiomed.2006.09.011 17112497

[18] Rattner DW Park AE. Advanced devices for the operating room of the future. Semin Laparosc Surg. (2003) 10(2):85–9. 10.1177/107155170301000205 12835831

[19] Mascagni P Padoy N. OR Black box and surgical control tower: recording and streaming data and analytics to improve surgical care. J Visc Surg. (2021) 158(3):S18–S25. 10.1016/j.jviscsurg.2021.01.004 33712411

[20] Shah N V Gold R Dar QA Diebo BG Paulino CB Naziri Q. Smart technology and orthopaedic surgery: current concepts regarding the impact of smartphones and wearable technology on our patients and practice. Curr Rev Musculoskelet Med. (2021) 14(6):378–91. 10.1007/s12178-021-09723-6 34729710 PMC8733100

[21] Pan S Zhang Z. Fundamental theories and basic principles of triboelectric effect: a review. Friction. (2019) 7(1):2–17. 10.1007/s40544-018-0217-7

[22] Suen SSH Khaw KS Wa Law L Sahota DS Lee SW Lau TK The force applied to successfully turn a foetus during reattempts of external cephalic version is substantially reduced when performed under spinal analgesia. J Matern Fetal Neonatal Med. (2012) 25(6):719–22. 10.3109/14767058.2011.589931 22043832

[23] Leung TY Sahota DS Fok WY Chan LW Lau TK. Quantification of contact surface pressure exerted during external cephalic version. Acta Obstet Gynecol Scand. (2003) 82(11):1017–22. 10.1034/j.1600-0412.2003.00269.x 14616275

[24] Kieweg SL Wilson SE Markovich G Simons S Manamendra HI Weiner CP. Biomechanics of birth – the fallacy of gentle birth: physician exerted pressures in vaginal and cesarean delivery. In: Lim CT Goh JCH, editors. 6th World Congress of Biomechanics (WCB 2010). August 1-6, 2010 Singapore. IFMBE Proceedings, Vol 31. Berlin, Heidelberg: Springer (2010). p. 683–5. 10.1007/978-3-642-14515-5_174

[25] Hodes A. The design and validation of a force and bending moment sensing device [Masters in bioengineering thesis]. (2015).

[26] Jones NW Mitchell EJ Wakefield N Knight M Dorling J Thornton JG Impacted fetal head during second stage Caesarean birth: a prospective observational study. Eur J Obstet Gynecol Reprod Biol. (2022) 272:77–81. 10.1016/j.ejogrb.2022.03.004 35290876

[27] Giblin L Vousden N Nathan H Gidiri F Goudar S Charantimath U Effect of the CRADLE vital signs alert device intervention on referrals for obstetric haemorrhage in low-middle income countries: a secondary analysis of a stepped- wedge cluster-randomised control trial. BMC Pregnancy Childbirth. (2021) 21:317. 10.1186/s12884-021-03796-4 33882864 PMC8061003

[28] Ngongo CJ Raassen T Lombard L van Roosmalen J Weyers S Temmerman M. Delivery mode for prolonged, obstructed labour resulting in obstetric fistula: a retrospective review of 4396 women in East and Central Africa. BJOG. (2020) 127(6):702–7. 10.1111/1471-0528.16047 31846206 PMC7187175

