# Supplementary material for: Preventing stillbirth from obstructed labor: A sensorized, low-cost device to train in safer operative birth
Source: Front Glob Womens Health. 2023 Jan 30;3:1039477. doi: 10.3389/fgwh.2022.1039477 (PMC9922699; doi:10.3389/fgwh.2022.1039477)
Supplement: Supplementary file 1 [file Datasheet1.docx]

**Preventing stillbirth from obstructed labour: a sensorized, low-cost device to improve safety and facilitate training in operative vaginal birth**

**Authors:** Shireen R. JAUFURAULLY (MBChB, BSc)^1,2^*, Carmen SALVADORES FERNANDEZ (MEng)^1,3^, Biswajoy BAGCHI (PhD)^1,3^, Priya GUPTA (PhD)^1,3^, Adrien DESJARDINS (PhD)^1,4^, Dimitrios SIASSAKOS (MD(Res) MBBS)^1,2,5^, Anna L. DAVID (PhD, MBChB)^1,2,5^, Manish K. TIWARI (PhD)^1,3*^.

1 Wellcome/EPSRC Centre for Interventional and Surgical Sciences, University College London, London, UK

2 Elizabeth Garrett Anderson Institute for Women’s Health, University College London, London, UK

3 Nanoengineered Systems Laboratory, Mechanical Engineering, University College London, London, UK

4 Department of Medical Physics and Biomedical Engineering, University College London, London, UK

5 National Institute for Health Research (NIHR) University College London Hospitals Biomedical Research Centre (BRC), London, UK

*Corresponding authors: Shireen Jaufuraully. Email: [s.jaufuraully@ucl.ac.uk](mailto:s.jaufuraully@ucl.ac.uk). Manish K Tiwari. Email: [m.tiwari@ucl.ac.uk](mailto:m.tiwari@ucl.ac.uk). Address: Wellcome/EPSRC Centre for Interventional and Surgical Sciences, University College London.

**Supplementary Information**

**1. Force calibration curves**

Each sensor was individually calibrated by plotting the force values against the measured resistance and fitting a power function to the data, as shown in Equation 1.

| F = a I + b  where F is force, I is the current measured from the sensor and a, b are the fit coefficients. |
| --- |

**Equation 1 Supplementary Information.** Translational linear function obtained through calibration relating measured current produced by the sensors to the force applied.

The linear function is chosen since it describes the behaviour of triboelectric sensors under compressive strain^1^. The approach yields a good fit for our sensors as well (see a particular example in Figure 1 - Supplementary Material and the corresponding power function shown in Equation 2 with an R-squared value of 0.95). Each sensor used for the glove was individually calibrated, and comprised slight variations in the fit coefficients of the equation relating measured current and force applied.

**Figure 1 Supplementary Information.** Plot obtained after the calibration test of a given sensor showing force applied against current change measured and linear function fit.

| F = 0.62 $\times$ I – 0.81  R^2^ = 0.95  where F is force, I is the current measured from the sensor and R^2^ is the R-squared value. |
| --- |

**Equation 2 Supplementary Information.** Translational linear function obtained for a particular sensor through calibration relating measured current produced by the sensors to the force applied.

**2. Fetal phantom development**


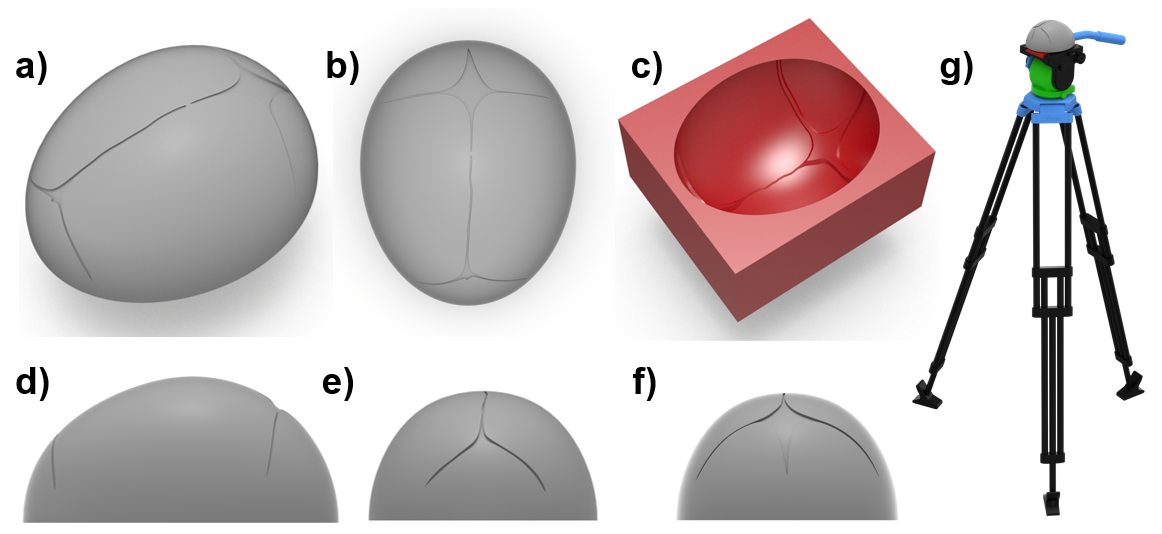


**Figure 2 Supplementary Information.** Initial phantom models and mold designed to replicate the fetal head with no molding or swelling. a) Phantom model of the fetal head. b) Top view phantom model. c) Mold created for the phantom model. d) Side view phantom model. e) Front view phantom model. f) Back view phantom model. g) Tripod setup to carry out the tests with the phantom models.

The first step when developing the phantoms was to create 3D computer-aided design (CAD) versions of the targeted phantom incorporating the sutures and fontanelles **(Figure 2a and 2b Supplementary Information)** and the mold **(Figure 2c Supplementary Information)** it would fit in. These were created using Autodesk Inventor. Once the molds were finalized, they were 3D-printed using Polylactic acid (PLA). Ecoflex, a silicone elastomer, was then poured into the fabricated molds and cured at 80 ºC for 2 h. The cured ecoflex phantom **(Figure 2a, 2b, 2d, 2e and 2f Supplementary Information)** were then extracted from the molds and fixed on the tripod setup to carry out the tests outlined in the manuscript.

The anterior fontanelle in term infants ranges from 6-36mm2, and the posterior fontanelle measures 5-7mm3. Molds were thus developed to give an anterior fontanelle size of 14mm and posterior fontanelle size of 5mm. There is a wide variation in suture widths reported in studies. One study provided measurements of 1.96-2.76mm depending on the suture4, and another reported sagittal suture width of 5.0 ± 0.2mm and a coronal suture width of 2.5 ± 0.1 mm5. The molds were created to give a suture diameter of 2.9mm.


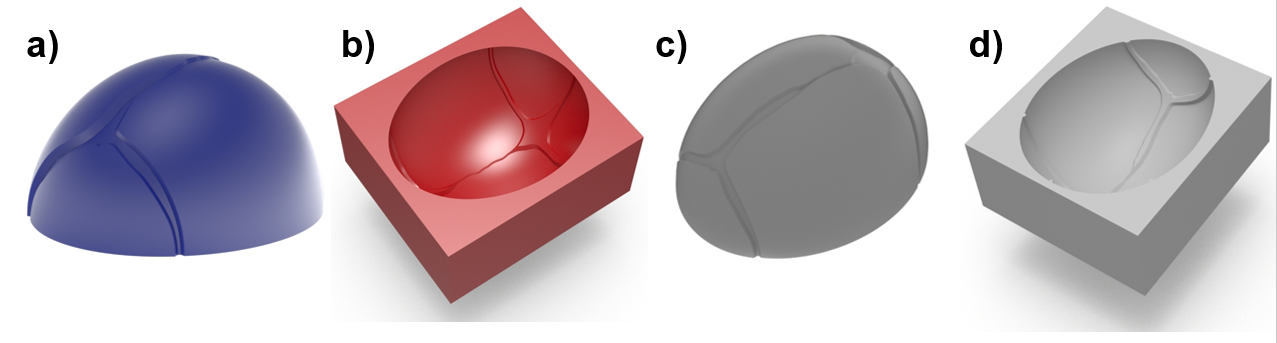


**Figure 3 Supplementary Information.** Phantom models and mold designed to replicate the fetal head with different degrees of molding.

Phantoms were also developed to replicate different degrees of molding by carrying out the procedure outlined above. For this purpose, new phantoms and molds were fabricated to accommodate for different levels of molding that may arise during labor **(Figure 3 Supplementary Information)**.

Finally, in order to reproduce swelling on the fetal heads, an additional mold was created **(Figure 4a Supplementary Information)** with a smooth finish (no sutures or fontanelles). This mold together with a cover for it with the initial phantom **(Figure 4a Supplementary Information)** attached were 3D-printed. Polydimethylsiloxane (PDMS) with induced air gaps was poured into the mold and the cover was assembled on to it **(Figure 4c Supplementary Information)** before curing for 2 h at 80 ºC. This was done in order to fabricate a thin layer surrounding the neonatal phantom **(Figure 4d Supplementary Information)** which was then extracted from the mold and fixed on any of the ecoflex phantoms previously described.


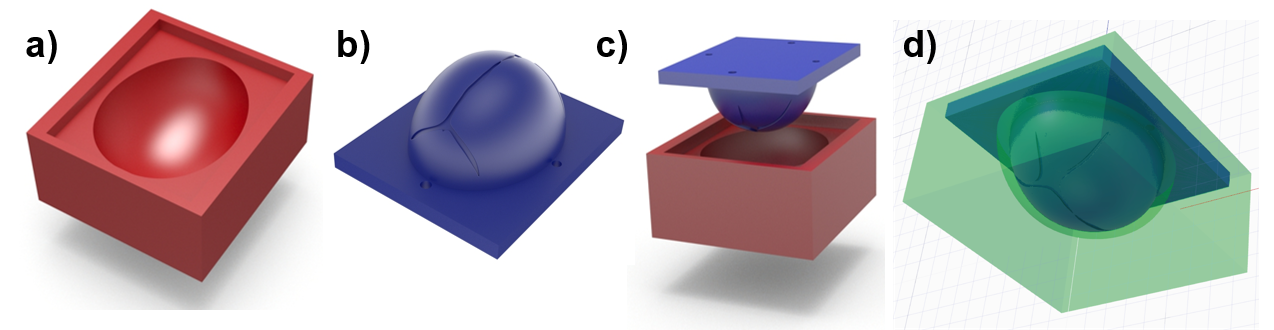


**Figure 4 Supplementary Information.** Phantom models and mold designed to replicate swelling on the fetal head.

**3. PPI panel video links**

**3.1 Sensor gloves for operative birth introduction to panel video**

https://www.youtube.com/watch?v=nFtTJbLPBYg

**3.2 Experience of shaping research with lived experience parents**

<https://www.youtube.com/watch?v=-K4OuxhyjmQ>

**References**

1. Pan S, Yin N, Zhang Z. Time-& load-dependence of triboelectric effect. Sci Rep. 2018;8(1):2-10. doi:10.1038/s41598-018-20937-6
2. Kiesler J, Ricer R. The abnormal fontanel. Am Fam Physician. 2003 Jun 15;67(12):2547-52. PMID: 12825844.
3. Popich GA, Smith DW. Fontanels: range of normal size. J Pediatr. 1972 May;80(5):749-52.
4. Riahinezhad M, Hajizadeh M, Farghadani M. **Normal Cranial Sutures’ Width in an Iranian Infant Population** Journal of Medical and Surgical Research. 2019;5(3):564-569.
5. Mitchell, C.A. Kitley, T.L. Armitage, M.V. Krasnokutsky, V.J. Rooks. Normal Sagittal and Coronal Suture Widths by Using CT Imaging. American Journal of Neuroradiology. 2011;32(10):1801-1805; **DOI:** 10.3174/ajnr.A2673
